# What’s Trending: Cross-Sectional Analysis of the Content and Quality of Popular Melanoma Videos on TikTok

**DOI:** 10.2196/79250

**Published:** 2026-08-25

**Authors:** Nazaneen Saleh, Alexa Rahaman, Emily Siegel

**Affiliations:** 1 Rush Medical College, Rush University Medical Center, 1620 W Harrison St, Chicago, IL, 60607, United States, 1 5712130078; 2 School of Medicine and Health Sciences, George Washington University, Washington, DC, United States

**Keywords:** dermatology, melanoma, social media, TikTok, trends

## Abstract

In this cross-sectional analysis of popular melanoma-related TikTok videos, physician-created, educational, and longer videos were associated with higher information quality and audience engagement, although overall DISCERN scores remained low and treatment guidance was frequently absent, highlighting opportunities to improve comprehensive, physician-led melanoma education on social media.

## Introduction

### Background

As social media continues to integrate into daily life, platforms like TikTok, which now exceeds 1.8 billion users, create opportunities to efficiently share engaging health information [1]. While this can enhance health literacy, it also raises concerns about misinformation.

TikTok has become a popular platform for dermatology content, with both physicians and nonexperts sharing advice. Given the 42% rise in invasive melanoma cases in the United States from 73,870 cases in 2015 to 104,960 estimated cases in 2025, health literacy and early detection are increasingly important for improving survival [2-4]. The dermatology literature has gaps in analyzing the widespread utilization of TikTok videos for delivering educational health information. To the best of our knowledge, this is the first study examining melanoma-specific videos on TikTok.

### Objective

This study aims to analyze the content and quality of popular melanoma-related videos on TikTok.

## Methods

TikTok was queried using the hashtags #melanoma, #metastaticmelanoma, and #skincancer on two pre-existing accounts. In total, 32 videos were collected by two authors in September 2024 and independently evaluated by dermatology residents and attendings at Rush University (Chicago, United States) using the DISCERN tool, a validated measure for assessing consumer health information [5]. The DISCERN questionnaire is divided into 3 parts: reliability of the publication (questions 1-8), quality of information about treatment options (questions 9-15), and the overall score of the publication (question 16). Each item is rated on a scale from 1 (poor) to 5 (excellent). An adjusted DISCERN score was calculated by averaging only the reliability-related items (questions 1‐8), since 28 of the 32 videos lacked treatment content. Only one video per TikTok creator was considered and every video was reviewed by both authors to ensure no repeated videos.

## Results

The content creators of the 32 videos consisted of physicians (n=18, 56%), nonphysicians (n=11, 34%), private companies (n=2, 6%), and organizations (n=1, 3%) (Table 1). Among the 18 physician creators, the majority were dermatologists (13/18, 72%). The mean DISCERN score across all videos was 2.08, highlighting the overall need for improved quality in melanoma-related content on TikTok. When treatment-related questions were excluded, the adjusted DISCERN score rose to 2.68, indicating that guidance on treatment is frequently lacking.

**Table 1.** Summary of TikTok video characteristics, quality scores, and engagement metrics.

Category and subgroup		Number of videos, N=32 (%)	Mean DISCERN score	Mean adjusted DISCERN score	Mean number of likes	Mean number of saves	Mean number of shares
Content creator type							
	Physician	18 (56)	2.3	2.98	17,499	2906	1525
	Dermatology	13/18 (72)	2.14	2.87	17,753	2388	1428
	Internal medicine	2/18 (11)	2.19	3.13	40,650	10,500	4351
	Emergency medicine	1/18 (6)	3.69	4.38	1889	85	95
	Oncology	1/18 (6)	2.44	2.5	661	167	83
	Surgery	1/18 (6)	2.94	3.25	357	16	7
	Nonphysician^a^	11 (34)	1.79	2.36	6515	1025	361
	Private company^b^	2 (6)	2.19	2.44	8997	2729	1155
	Nonprofit organization^c^	1 (3)	1.19	1.38	2518	994	165
Video length							
	<1 minute	17 (53)	2.01	2.57	3973	768	313
	>1 minute	15 (47)	2.15	2.81	22,642	3799	1906
Video type							
	Anecdotal	7 (22)	1.72	2.13	5813	584	320
	Educational	25 (78)	2.18	2.84	14,659	2638	1267

a Nonphysician: personal TikTok accounts run by any individuals who were not physicians.

b Private company: accounts promoting or selling products with a for-profit purpose.

c Nonprofit organization: organization affiliated with Oregon Health & Science University Dermatology (Start Seeing Melanoma).

## Discussion

The analysis of content creators revealed that among the 18 physician creators, a significant majority (n=13, 72%) were dermatologists. This was expected as dermatology is the specialty with the most training in diagnosing and treating melanoma. However, the mean DISCERN score for videos created by dermatologists was 2.19, compared to 3.69 for emergency medicine physicians, indicating a general need for improved quality in melanoma-related content on TikTok regardless of physician training. Notably, when treatment-related questions were excluded, the adjusted DISCERN score increased from 2.08 across all videos to 2.68, suggesting that there is a frequent absence of guidance regarding treatment options in the content. Enhancing content with accurate, treatment-focused information may improve health literacy among viewers.

Physician-created videos outperformed those by nonphysicians in both quality (DISCERN: 2.3 vs 1.79, respectively; adjusted: 2.98 vs 2.36, respectively) and audience engagement metrics (mean number of likes: 17,499 vs 6515, respectively). This underscores the importance of professional involvement in online health education. Similarly, videos longer than one minute scored higher than shorter ones (DISCERN: 2.15 vs 2.01; adjusted: 2.81 vs 2.57), and educational videos outperformed anecdotal content (DISCERN: 2.18 vs 1.72; adjusted: 2.84 vs 2.13). Longer and educational videos also showed greater audience engagement. Together, these findings suggest that TikTok’s short-form format does not inherently disadvantage content that prioritizes explanation and educational intent. Rather than implying viewer preference for longer videos, the observed associations indicate that informational clarity and depth can coexist with algorithm-driven visibility on the platform.

Limitations include the study’s cross-sectional design, use of only three hashtags, platform-specific sample, and inability to verify creator credentials. Additionally, TikTok’s proprietary search algorithm may affect the visibility and discoverability of videos among different users, introducing an element of unpredictability. Lastly, the DISCERN tool was originally developed to assess written health information and may not fully capture the unique elements of video-based content. Future research should explore tools tailored to assess video content and investigate strategies for optimizing physician-led education on social media platforms.
